# New Method of Expelling Foreign Bodies

**Published:** 1849

**Authors:** 


### ARTICLE V:

### NEW METHOD OF EXPELLING FOREIGN BODIES.

Dr. Charles Hansford, of Knoxville, in this State, gives us the following history of a case, illustrative of the efficiency of a new method of expelling foreign substances from the larynx.

A colored girl had accidentally got a pin into the windpipe and was suffering with all the distressing symptoms consequent upon obstruction of the air passages from a foreign body.

She was directed to lie upon a bench, face downwards with the head projecting over the edge, and to take a full inspiration.

Whilst in this position, with the lungs filled with air, a smart blow or two on the back with a pillow, made hard and firm by compression, had the effect to expel the pin at once from the larynx. In this case, the first blow moved the pin about an inch, the second forced it into the mouth.

"Since that time," says our correspondent, "I have had several opportunities of trying the maul made of a pillow. I have driven out water-melon seeds in this manner, on three different occasions; a grain of corn at one time, and a large glass bead at another."

The treatment recommended above is ingenious and simple, and, as it seems to us, is worthy of being tried in cases like those cited above. H.

